# Extremely high-gain source-gated transistors

**DOI:** 10.1073/pnas.1820756116

**Published:** 2019-02-25

**Authors:** Jiawei Zhang, Joshua Wilson, Gregory Auton, Yiming Wang, Mingsheng Xu, Qian Xin, Aimin Song

**Affiliations:** ^a^School of Electrical and Electronic Engineering, University of Manchester, Manchester M13 9PL, United Kingdom;; ^b^National Graphene Institute, University of Manchester, Manchester M13 9PL, United Kingdom;; ^c^State Key Laboratory of Crystal Materials, Centre of Nanoelectronics and School of Microelectronics, Shandong University, Jinan 250100, People’s Republic of China

**Keywords:** source-gated transistor, oxide semiconductors, Schottky barrier, inhomogeneities, intrinsic gain

## Abstract

Despite being a fundamental electronic component for over 70 years, it is still possible to develop different transistor designs, including the addition of a diode-like Schottky source electrode to thin-film transistors. The discovery of a dependence of the source barrier height on the semiconductor thickness and derivation of an analytical theory allow us to propose a design rule to achieve extremely high voltage gain, one of the most important figures of merit for a transistor. Using an oxide semiconductor, an intrinsic gain of 29,000 was obtained, which is orders of magnitude higher than a conventional Si transistor. These same devices demonstrate almost total immunity to negative bias illumination temperature stress, the foremost bottleneck to using oxide semiconductors in major applications, such as display drivers. Furthermore, devices fabricated with channel lengths down to 360 nm display no obvious short-channel effects, another critical factor for high-density integrated circuits and display applications. Finally, although the channel material of conventional transistors must be a semiconductor, by demonstrating a high-performance transistor with a semimetal-like indium tin oxide channel, the range and versatility of materials have been significantly broadened.

## Significance

The advent of thin-film electronics and the development of new materials, such as oxide semiconductors, bring great opportunities for new device designs and applications. A source-gated transistor (SGT) is created by combining two fundamental building blocks of electronics: a thin-film transistor and a Schottky diode. By developing a methodology to manipulate the source barrier profile, an extremely high gain of 29,000 is achieved, previously unthinkable with a single component. The SGT design overcomes several major bottlenecks to using oxide semiconductors in large-scale applications and broadens the range of channel materials beyond standard semiconductors. These advantages combined with an analytical theory confirmed by simulations and experiments demonstrate the potential of SGTs to be a fundamental component for thin-film electronics.

Transistors are the bedrock of the recent technology revolutions that have shaped the modern world. To drive further advancement, new transistors must be designed to meet industry needs. One unconventional transistor design combines the thin-film transistor (TFT) with another fundamental component of electronics, the Schottky diode (1). The resulting advantages include high intrinsic gain (2–4), low-voltage saturation (5), insensitivity to channel length and semiconductor quality (1, 6), and improved stability (7). Within the literature, such devices with common designs and characteristics are given various names, such as source-gated transistors (SGTs), Schottky barrier TFTs, and tunneling contact transistors. Under these different names, conflicting theories of device operation continue to be put forward. For example, the gate dependence of the current has been variously attributed to lowering of the source barrier height (8), increased tunneling current (4), and modulation of the effective source length (9). There are also differing claims about the effects of using a Schottky drain contact (4, 9). Similarly, diode reverse current saturation (4), tunneling (10), and depletion of the semiconductor by the source (5) have all been suggested as causes of current saturation.

The SGT structure is particularly advantageous for thin-film electronics, where oxide semiconductors have opened a new era in microelectronics, particularly for large-area, flexible, and transparent applications (11–14). The wide bandgap of oxide semiconductors (typically >3 eV) allows for high optical transparency, while room temperature processability offers compatibility with flexible substrates. Although oxide semiconductors, particularly indium–gallium–zinc oxide (IGZO), are nearing maturity (15–18), there remain major barriers to large-scale adoption. For example, when IGZO TFTs are held at negative gate bias and elevated temperature and illuminated with near-bandgap energy photons, there is a negative shift in the turn-on voltage (19, 20). This susceptibility to negative bias illumination temperature stress (NBITS) is one of the main factors delaying the wide-scale adoption of IGZO in the display industry. Another major problem is that TFTs require precise lithography to enable large-area uniformity, and difficulties with registration between different TFT layers are likely to be compounded by the use of flexible substrates. One major advantage of the SGT is that it does not require such precise registration, because the current is controlled by the dimensions of the source contact rather than the channel. Moreover, recent work on ultralow-power oxide semiconductor SGTs has yielded devices with an intrinsic gain of 400, far greater than standard oxide semiconductor TFTs and conventional Si transistors (4). Such high-gain devices may find application in analogue circuits, sensors, and large-area displays.

In this work, TFTs exhibiting extremely high gain are designed using our recent discovery of the conduction mechanism in reverse-biased thin-film Schottky diodes (21). Based on these designs and derived analytical theory, oxide semiconductor TFTs with intrinsic gains consistently above 10,000 (peaking around 29,000) are demonstrated. Furthermore, we are able to produce oxide semiconductor TFTs that are intrinsically impervious to NBITS. Moreover, these same devices show no indication of performance degradation down to channel lengths of 360 nm, one or two orders of magnitude smaller than typical IGZO TFTs (22, 23). Finally, our design no longer restricts the channel layer to being a semiconductor as demonstrated by using a semimetal-like oxide, indium tin oxide (ITO).

## Results

### SGTs.

**Fig. 1.**
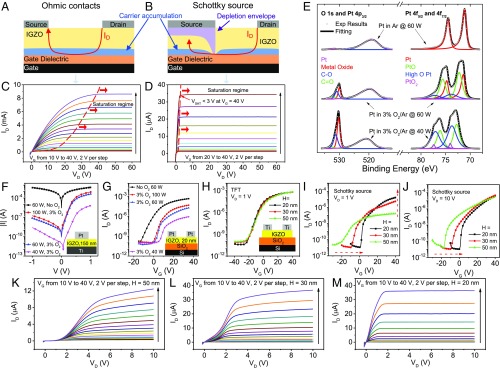
Designing and optimizing SGTs through tuning source contact deposition conditions and semiconductor thickness. (*A*) Structure and conduction path in a TFT with ohmic contacts. (*B*) Structure and conduction path in a TFT with Schottky contacts showing how the current saturates due to depletion under the source. (*C* and *D*) Typical output curves for a TFT (*C*) and an SGT (*D*); the significant difference in saturation voltage occurs because the SGT is so easily depleted beneath the source. (*E*) XPS results for Pt films sputtered at 60 W in Ar (*Top*), 60 W in $3\%\;{\text{O}}_{2}$/Ar (*Middle*), and 40 W in $3\%\;{\text{O}}_{2}$/Ar (*Bottom*). (*F*) |I|−V curves for Pt–IGZO Schottky diodes with different powers and oxygen contents during Pt deposition (device structure in *Inset*). (*G*) Transfer curves for Pt–IGZO SGTs with different powers and oxygen contents during Pt deposition (device structure in *Inset*). (*H–J*) Transfer characteristics displaying the thickness dependence of IGZO TFTs (*H*) at $V_{D}=1$ V (device structure in *Inset*), SGTs at $V_{D}=1$ V (*I*), and SGTs at $V_{D}=10$ V (*J*). (*K–M*) Output characteristics for SGTs with 50-nm (*K*), 30-nm (*L*), and 20-nm (*M*) IGZO thickness. Fabricated TFTs and SGTs have source lengths =1.2 mm and channel lengths =60 μm.

A conventional TFT comprises source and drain electrodes, which are joined by a semiconductor channel. For the TFT to operate, the contacts should be ohmic (i.e., of low resistance). The channel is capacitively coupled to a gate electrode via an insulating dielectric, and thus, the gate voltage, $V_{G}$, controls the conductivity of the channel (Fig. 1*A*). In SGTs (Fig. 1*B*), the source contact is replaced by a diode-like Schottky barrier. As such, it is the source rather than the channel that determines the current (5, 9).

The effect of replacing the ohmic source with a Schottky source is demonstrated by the output curves of an IGZO TFT and SGT in Fig. 1 *C* and *D*. The TFT current only saturates at high drain voltages, whereas the saturation at significantly lower voltages in the SGT is made possible by the full depletion of the semiconductor layer by the Schottky source (Fig. 1*B*) (5, 9). More importantly, the better saturation in the SGT means that the intrinsic gain, which is a critical figure of merit for transistors, far exceeds that of a TFT.

### Source Barrier Control.

The barrier at the source is the most important feature of the SGT. Forming a Schottky source on oxide semiconductors is highly challenging and dependent on having sufficient oxygen content at the interface (24–26). The need for a conductive channel further complicates fabrication, as oxygen vacancies are the donor states in oxide semiconductors (27). Moreover, postannealing to improve conductivity can damage the barrier (28), and therefore, the annealing required to produce a conductive channel must be carried out before the deposition of the Schottky contact. Thus, to guarantee sufficient oxygen at the interface, oxygen was included during the deposition of the Schottky contact. Sputtering Pt in $3\%\;{\text{O}}_{2}$/Ar and controlling the deposition power allowed us to control the oxygen content in the Pt film. X-ray photoelectron spectroscopy (XPS) results in Fig. 1*E* show negligible oxygen content in the Pt film deposited in pure Ar. When oxygen is introduced, the ratio of O 1s to Pt ${4\text{p}}_{3/2}$ peak areas grows, and the Pt ${4\text{f}}_{5/2}$ and ${4\text{f}}_{7/2}$ peaks shift to the left, indicating oxidation of the Pt film. The oxygen content is further increased by reducing the sputtering power from 60 to 40 W. Additional discussion of the XPS results can be found in *SI Appendix*.

The suitability of Schottky contacts with different oxygen contents was tested by fabricating Pt–IGZO Schottky diodes and SGTs. Fig. 1*F* shows the I−V curves for the Schottky diodes, and Fig. 1*G* shows the SGT transfer curves. Without oxygen treatment, the Pt–IGZO diode is effectively ohmic due to the formation of lower barrier regions in the Schottky contact. The lower barrier regions may be due to ${\text{In}}^{3+}$ being reduced to ${\text{In}}^{0}$ as a result of insufficient oxygen at the interface (29). Using oxygen-rich Pt as a contact lowers the reverse current in the diode and on current in the SGT by making the barrier more homogeneous. The fact that some barrier height inhomogeneities remain is evidenced by the strong bias dependence of the diode reverse current (21) and low-temperature measurements (*SI Appendix*, Fig. S1 *A* and *B*). Varying the sputtering power also affects the barrier inhomogeneities, as the barrier height extracted from the diode I−V curves falls with increasing sputtering power (*SI Appendix*, Fig. S1*C*). Higher sputtering power leads to a faster Pt deposition rate, and therefore, less oxygen is incorporated at the Pt–IGZO interface. As a result, increasing power causes an increase in the on current and a reduction in turn-on voltage of the SGTs. Although using a deposition power of 100 W gives a slightly higher on current in the SGT, a power of 60 W gives a more consistent barrier height, and therefore, it was selected as the optimal condition for Pt deposition. Additional details about the effects of deposition conditions on the barrier are shown in *SI Appendix*, Fig. S1.

### Semiconductor Thickness Dependence.

Recently, we have shown a dramatic dependence of the reverse current of Schottky diodes on semiconductor thickness (21). Thus, by tuning the thickness, we may be able to optimize the SGT operation. To test this hypothesis, TFTs and SGTs with 20-, 30-, and 50-nm-thick IGZO layers were fabricated simultaneously (statistical analysis of the transfer curves of the 20-nm SGT is in *SI Appendix*, Fig. S2). As expected, the TFTs showed no discernible thickness dependence (Fig. 1*H*). By contrast, the SGT transfer curves in Fig. 1 *I* and *J* show two strong thickness dependencies. First, when the drain voltage, $V_{D}$, is 10 V, the turn-on voltage, $V_{ON}$, increases from −18 V in the 50-nm case to 0 V in the 20-nm case. The modulation of $V_{ON}$ can be attributed to the ease of channel depletion by the Schottky source; thinner semiconductors are more easily depleted and hence, require a more positive $V_{G}$ to turn the channel on. Second, seemingly counterintuitively, thinner devices in Fig. 1*I* have a greater on current, which is not fully explained by the literature (30).

Two more trends that cannot be fully explained with existing understanding are present in output curves in Fig. 1 *K*–*M*. First, a thinner semiconductor gives more linear curves at low $V_{D}$. Second and critically, in the saturation region where the device is operated, a thinner semiconductor gives a flatter and therefore, more desirable saturation. The flatness of saturation current is particularly important for achieving high intrinsic gain. Strikingly and somewhat surprisingly, an increase in gain of nearly two orders of magnitude is observed when the IGZO thickness is reduced from 50 to 20 nm. To investigate the origins of this sensitive thickness dependence, device simulations were carried out.

### Effects of Barrier Inhomogeneities.

**Fig. 2.**
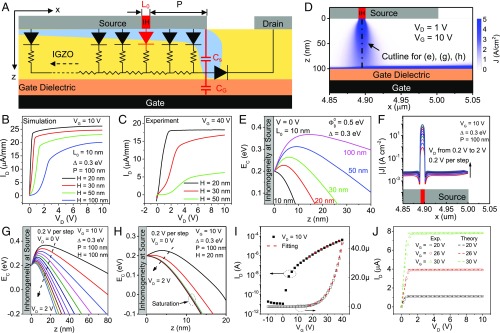
Simulations and theory for SGTs. (*A*) The distributed model for SGTs overlaid on the simulated device structure showing the barrier height inhomogeneity (IH; in red). (*B* and *C*) Output curves displaying the semiconductor thickness dependence of the SGT in device simulations (*B*) and experiments (*C*). In *B*, the mean barrier height, $\Phi_{B}^{0}$, is 0.5 eV, and the barrier height at the inhomogeneity is $\Phi_{B}=\Phi_{B}^{0}-\Delta =0.2$ eV. The inhomogeneity width, $L_{0}$, is 10 nm, and the distance from the source edge, P, is 100 nm. (*D*) Current density distribution in the SGT in *B* with 100-nm-thick IGZO when $V_{G}=10$ V and $V_{D}=1$ V. (*E*) Profiles of the conduction band minimum beneath the inhomogeneity at zero bias for different thicknesses. (*F*) Profiles of current density across the source in *D* for $V_{D}=0-2$ V. (*G* and *H*) Profiles of the conduction band minimum beneath the center of the inhomogeneity of the SGT in *B* with 100-nm-thick (*G*) and 20-nm-thick (*H*) IGZO for $V_{D}=0-2$ V. (*I* and *J*) Fitting of the measured transfer curve when $V_{D}=10$ V (*I*) and the measured output curves when $V_{G}=$20, 26, and 30 V (*J*). Fabricated devices have source lengths =1.2 mm and channel lengths =60 μm; simulated devices have source lengths =5 μm and channel lengths =2 μm.

SGTs were simulated in Silvaco Atlas with a barrier inhomogeneity (a region of lower barrier height) inserted into the Schottky source contact as shown in Fig. 2*A*. The thickness dependencies seen in the experiments are clearly replicated by the simulations as depicted in Fig. 2 *B* and *C*. The current distribution in Fig. 2*D* shows that the current is dominated by the contribution from the lower barrier inhomogeneity. Fig. 2*E* compares the profiles of the conduction band minimum along the vertical dashed line in Fig. 2*D* from the center of the inhomogeneity at zero bias for different semiconductor thicknesses. For thicker semiconductor layers, a saddle point is established beneath the inhomogeneity due to depletion by the surrounding higher barrier regions. As the IGZO is made thinner, the electric field increases and reduces the saddle point height until, at a certain thickness, it is removed entirely (21). Although the existence or absence of a saddle point does not affect the saturation voltage, the removal of the saddle point (or at least, the alleviation of its effects) is the key to obtaining high gain as discussed below.

To establish how the saddle points affect the $I_{D}-V_{D}$ relation, profiles of the current density along the Schottky interface were taken for an IGZO thickness, H, of 100 nm (Fig. 2*F*). Unlike the rest of the source, the current through the inhomogeneity increases exponentially by two orders of magnitude as $V_{D}$ increases from 0.2 to 2 V. The origin of the exponential growth is elucidated in Fig. 2*G*, which shows the strong voltage dependence of the saddle point, amounting to a voltage-dependent effective barrier height. When H = 20 nm, as in Fig. 2*H*, the saddle point is much lower at zero bias, and more importantly, it has a much weaker bias dependence; hence, there is no exponential $I_{D}-V_{D}$ relation at low $V_{D}$. After it is saturated, it is the absence of a saddle point that enables much flatter current saturation and therefore, the striking two orders of magnitude increase in gain. A full understanding the role of barrier height inhomogeneities in SGTs is particularly important for enabling the design of devices using disordered materials. More simulations and discussions are found in *SI Appendix*, Figs. S3 and S4.

### Theoretical Analysis of SGTs.

Other than simulations, an analytical theory can be derived to allow for additional understanding of device behavior (full derivation is in *SI Appendix*, Fig. S5). In high-gain devices, the saddle points no longer have a significant effect, and the effective barrier height should take the form $\Phi_{B,eff}=\Phi_{B}^{0}-\Phi_{IFL}-\alpha qE_{M}$, where $\Phi_{B}^{0}$ is the mean barrier height and $\Phi_{IFL}$ and $\alpha qE_{M}$ are barrier-lowering terms due to the image force effect and the electric field, respectively (31). In an SGT, most of the current passes through the front end of the source, and our detailed analysis shows that the current in the linear regime can be written as

$$\begin{array}{ll} I_{lin} & =W\sqrt{\frac{qN_{C}C_{G}\left(V_{G}-V_{T}\right)e^{-\Phi_{B,ef\;f}/kT}}{H}}\\ & \times \mu_{n}\left(\frac{\Phi_{B}^{0}}{q}+V_{D}\right)\left(1-e^{-qV_{D}/kT}\right). \end{array}$$ [1]

Similarly, in the saturation regime, the current is given by

$$\begin{array}{ll} I_{sat} & =W\sqrt{\frac{qN_{C}C_{G}\left(V_{G}-V_{T}\right)e^{-\Phi_{B,ef\;f}/kT}}{H}}\\ & \times \mu_{n}\left[\frac{C_{G}}{C_{S}+C_{G}}\left(V_{G}-V_{T}\right)+\frac{\Phi_{B}^{0}}{q}\right], \end{array}$$ [2]

where W is the source contact width, q is the fundamental charge, $\mu_{n}$ is the electron mobility in the semiconductor, $N_{C}$ is the effective density of states in the conduction band, $V_{T}$ is the threshold voltage of the SGT, k is the Boltzmann constant, T is the temperature, and $C_{S}$ and $C_{G}$ are the capacitance per unit area of the semiconductor and the capacitance per unit area of the gate insulator, respectively. In this experiment, $\mu_{n}=10.6$ ${\text{cm}}^{2}$/Vs (obtained from an IGZO TFT), W = 2 mm, and the relative permittivity is 3.9 for ${\text{SiO}}_{2}$ and 10 for IGZO. The experimental transfer curve (dots) in Fig. 2*I* shows a very good agreement with the values obtained from Eq. **2** (dashes in Fig. 2*I*). The fitting also yields α=0.73 nm, $V_{T}$ = 11.7 V, and $\Phi_{B}^{0}=0.74$ eV, which agree almost perfectly with the results for barrier height in *SI Appendix*, Fig. S1*C*. Using these same parameters, the output curves also agreed very well with the theory (Fig. 2*J*). The above results indicate that our analytical formulas offer an accurate description of the I−V characteristics of an SGT.

### Extremely High Intrinsic Gain.

**Fig. 3.**
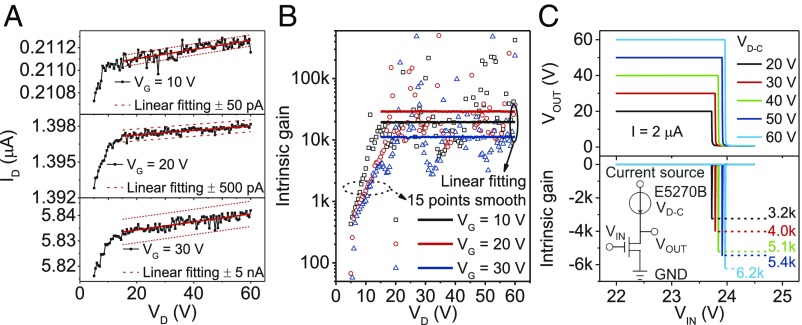
Intrinsic gain measurements. (*A*) Zoomed output curves of the SGTs with 20-nm-thick IGZO for $V_{G}=$10, 20, and 30 V. A linear fitting of the raw data is taken, as the very small fluctuations in current fall within the tolerance of the measurement equipment. (*B*) Intrinsic gain of the SGTs with 20-nm-thick IGZO for $V_{G}=$10, 20, and 30 V. The intrinsic gain values obtained by both the linear fitting and a 15-point smoothing of the output curves are displayed. (*C*) Intrinsic gain measured using an inverter with a current source as a load. The measurement setup is shown in *Inset*. SGTs have source lengths =1.2 mm and channel lengths =60 μm.

The intrinsic gain, $A_{v}$, is the maximum voltage gain of a transistor; thus, it is an important measure of a transistor’s ability to amplify a signal. $A_{v}$ can be calculated as the ratio of transconductance, $g_{m}=\partial I_{D}/\partial V_{G}$, to output conductance, $g_{d}=\partial I_{D}/\partial V_{D}$. As revealed in device simulations, the intrinsic gain in our SGTs is extremely high due to the removal or near removal of saddle points in the conduction band minimum. However, extracting the intrinsic gain directly from the I−V characteristics of our SGTs is extremely challenging due to the unprecedented flatness of the output curves. Such flatness requires highly precise measurement of minute changes in $I_{D}$ down to the very limit of our measurement setup resolution. The output curves (for the SGT with 20-nm-thick IGZO) in Fig. 3*A* demonstrate changes in current as low as a few picoamperes over a wide range of $V_{D}$ from 15 to 60 V. The solid red lines in Fig. 3*A* are linear fittings of the results between 15 and 60 V, and the dashed lines in Fig. 3*A* are a guide to the extent of the fluctuation.

Intrinsic gains of 19,000, 29,000, and 11,000 were obtained for $V_{G}=10$, 20, and 30 V, respectively, using the linear fittings in Fig. 3*A*. Using 15-point smoothing (Savitzky–Golay) of the output curves, the obtained gain values have good agreement with the linear fitting results, with some of the gain values even higher than 100,000 at certain biases (Fig. 3*B*). To further confirm the extremely high gain, the SGT was connected in an inverter setup using a current source as a load (Fig. 3*C*, *Inset*). The abrupt inversion gives a gain of 6,200, which is only limited by a drain compliance ($V_{D-C}$) of 60 V. With careful threshold voltage matching, perhaps by tuning semiconductor thicknesses, SGT-based inverters can have high noise margins (32).

As accurate measurement is difficult, the intrinsic gain of SGTs can only act as a figure of merit; however, our results represent an increase of nearly two orders of magnitude over other oxide semiconductor devices (4, 23, 33), suggesting a marked improvement in real-world performance. Transistors with such extremely high gain offer a potentially huge improvement on contemporary amplifiers and enable better circuit stability and signal to noise ratio, both of which are desirable for sensing weak signals (34). These advantages make the SGT highly suitable for application in the wearable and implantable health care devices that are expected to enter the market as part of the internet of things. The flat saturation of the SGT also makes it an excellent candidate for use as a current source in pixel circuits, as it provides a stable current over a wide range of $V_{D}$ (35).

### Short-Channel Effects.

To achieve high integration densities, transistor dimensions must be scaled down, but the short-channel effect has been the main obstacle to such scaling. In the case of IGZO TFTs, reducing the channel length below 5 μm produces a high-enough electric field to make the saturation current strongly dependent on $V_{D}$ (22, 23). In comparison, SGTs are more resilient to the short-channel effect, because the source region determines the current rather than the channel and its dimensions (1, 6, 36).

**Fig. 4.**
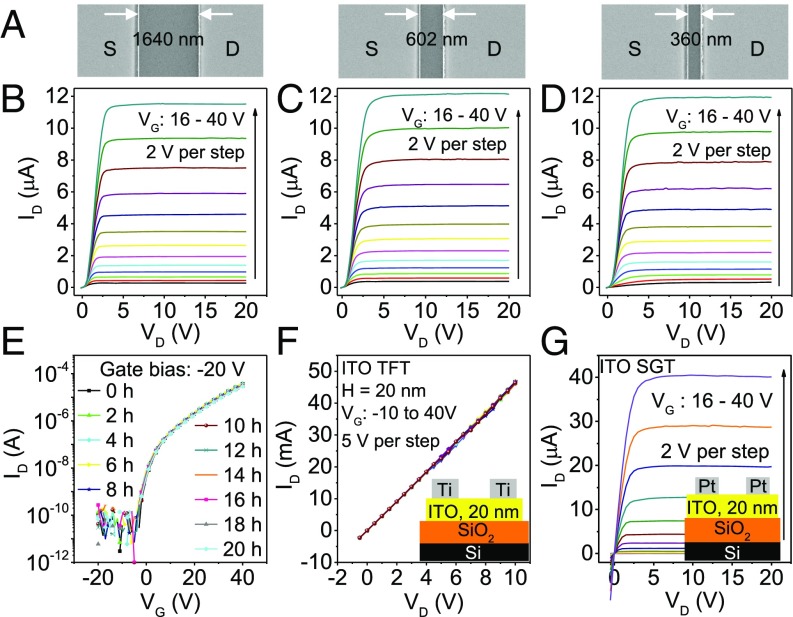
Advantages of SGTs for oxide materials. (*A*) SEM images of channel length of three short-channel SGTs. (*B–D*) Output curves for short-channel Schottky contact transistors with channel lengths of 1,640 nm (*B*), 602 nm (*C*), and 360 nm (*D*). None of the devices are affected by short-channel effects. (*E*) Transfer curves showing the device behavior under NBITS for 20 h. The device was exposed to heating at 60 °C and a 2,000-lx white LED, and it was biased at $V_{G}=-20$ V. (*F*) Output curves for a TFT with an ITO channel. (*G*) Output curves for an SGT with an ITO channel. Short-channel SGTs devices have source lengths =200 μm; all other SGTs have source lengths =1.2 mm and channel lengths =60 μm.

Using electron beam lithography, we fabricated IGZO SGTs with channel lengths of 360, 602, and 1640 nm. SEM images of the three channels are shown in Fig. 4*A*. Fig. 4 *B*–*D* shows that flat saturation up to $V_{D}=20$ V is maintained down to channel lengths of 360 nm. To the best of our knowledge, such an immunity to the short-channel effect has never been demonstrated with oxide semiconductors. Moreover, the current is highly consistent regardless of channel length, meaning that the SGTs are tolerant to alignment variations, which is of great importance to large-area electronics.

### NBITS.

NBITS is another long-standing barrier to the commercial application of oxide semiconductor TFTs (19, 20). Exposure to a combination of near-bandgap illumination, negative bias, and elevated temperature, as would be expected in a display circuit, produces a negative shift in the turn-on voltage of IGZO TFTs. NBITS tests were carried out on our 20-nm-thick IGZO SGTs. The devices were held at $V_{G}=-20$ V and 60 °C under illumination from a 2,000-lx white LED. Despite 20 h of stress, the device exhibited no discernible shift in $V_{ON}$ as shown in Fig. 4*E*. This high stability can be attributed to independence of the current from the channel conductivity. The high resistance of the source region will mask any channel instability. The immunity to NBITS removes a lasting obstruction to wide deployment of oxide semiconductors in the display industry.

### Other Oxide Materials.

The understanding of the working principle and design methodology in this work even removes the usual restriction that the channel layer can only be a semiconductor. Here, a semimetal-like oxide ITO is tested. The use of such a material is difficult in ordinary TFTs as shown by the lack of gate modulation in the ITO TFT (Fig. 4*F*). However, the output characteristics of an ITO SGT, as shown in Fig. 4*G*, are comparable with those of the IGZO SGT in Fig. 1*M*. The ITO SGT demonstrates that our Schottky source contact design can broaden the range of materials used for channel layers.

## Summary

By gaining a deeper understanding of the device physics of SGTs, particularly the control of the source barrier, we have, to the best of our knowledge, achieved an unprecedented gain in TFTs. Furthermore, the same devices are rendered immune to two of the most critical issues facing oxide transistors in industry: NBITS and the short-channel effect. The techniques used to achieve these results are applicable to all oxide semiconductors and even oxide conductors, thereby expanding the range of materials available for use as transistor channels. Most importantly, the underlying design principles and analytical theory of the SGT are applicable to all material types. As such, these devices have huge potential for applications in large-area displays, wearable and implantable sensors, and analogue circuits.

## Materials and Methods

All thin-film layers were deposited on ${\text{SiO}}_{2}$–Si wafers with 100-nm-thick ${\text{SiO}}_{2}$ using radiofrequency sputtering. For the Schottky diodes, a Ti layer was deposited as an ohmic contact followed by an IGZO layer. The samples were then annealed at 300 °C in ${\text{N}}_{2}$ for 1 h before the deposition of the Pt Schottky contact. For the SGTs, IGZO (or ITO) layers were deposited and annealed at 300 °C in ${\text{N}}_{2}$ for 1 h before the deposition of Pt source-drain contacts. For the IGZO TFTs, Ti was used as source-drain contacts.

The I−V characteristics were measured using a Keysight E5270B semiconductor analyzer at room temperature. The bias stress measurement was carried out on the Advanced Research Systems DE-204 temperature-controlled stage. Device simulations were carried out using Silvaco Atlas. XPS measurements were carried out using an Axis Ultra Hybrid. Full details are provided in *SI Appendix*.

## Supplementary Material

Supplementary File
