# Supplementary material for: Extremely high-gain source-gated transistors
Source: Proc Natl Acad Sci U S A. 2019 Feb 25;116(11):4843–8. doi: 10.1073/pnas.1820756116 (PMC6421470; doi:10.1073/pnas.1820756116)
Supplement: Supplementary File [file pnas.1820756116.sapp.pdf]

Supplementary Information for Extremely-High-Gain Source-Gated Transistors

Jiawei Zhang, Joshua Wilson, Gregory Auton, Yiming Wang, Mingsheng Xu, Qian Xin and Aimin Song*

* Corresponding Author Email: a.song@manchester.ac.uk

This PDF file includes:

- Materials and methods
- Supplementary text
- Figs. S1 to S5
- References for SI reference citations

www.pnas.org/cgi/doi/10.1073/pnas.1820756116

Materials and Methods

Fabrication of Schottky Diodes

Pt-IGZO Schottky diodes were fabricated using Ti as an ohmic contact. SiO_2-Si wafers were cleaned by sonic agitation in an ultrasonic bath using DECON 90, de-ionized water, acetone and isopropyl alcohol, respectively. Using radio-frequency (RF) sputtering of a Ti target, a 70 nm thick Ti layer was deposited. The working gas was Ar, the pressure was 5×10^{-3} mbar and the sputtering power was 150 W. A 150 nm thick IGZO layer was deposited via RF sputtering using an IGZO target with a molar ratio of 1:1:2 (In_2O_3:Ga_2O_3:ZnO). The working gas was Ar, the pressure was 5×10^{-3} mbar and the sputtering power was 100 W. Prior to Pt deposition the structure was annealed at 300 °C in a N_2 atmosphere for 1 hour. A 70 nm Pt layer was also deposited by RF sputtering a Pt target in either pure Ar or 3% O_2/Ar mix at a pressure of 5×10^{-3} mbar and the sputtering power was 60 W, unless otherwise stated. The devices were patterned using shadow masks.

Fabrication of Transistors

SGTs and TFTs were fabricated using SiO_2-Si wafers with 100 nm thick SiO_2. The wafers were cleaned by sonic agitation in an ultrasonic bath using DECON 90, de-ionized water, acetone and isopropyl alcohol, respectively. For the IGZO SGTs, IGZO and Pt layers were deposited using the same methods as for the Schottky diode and prior to Pt deposition the samples were annealed at 300 °C in a N_2 atmosphere for 1 hour. The IGZO TFTs, were the same as the SGTs, but Ti source and drain contacts were used

instead of Pt and were sputtered in the same way as for the Schottky diodes. Except for the channel layer, ITO SGTs were fabricated in a similar manner to the IGZO SGTs; the ITO target was sputtered in Ar, at a pressure of 5×10^{-3} mbar and a sputtering power of 100 W. The devices were patterned using shadow masks, except for the short-channel devices which were patterned using standard e-beam lithography.

Measurement of Device Characteristics

The standard I-V characteristics of all devices were measured using a Keysight E5270B semiconductor analyser at room temperature. For the calculation of intrinsic gain, the output curves of IGZO SGT were measured using a Keysight E5270B in pulsed mode with a period of 600 ms and averaged for 30 times. The low temperature measurements of IGZO Schottky diodes were carried out using a Lakeshore cryogenic CRX-4K probe station. The SEM images were taken using a Zeiss Sigma field emission scanning electron microscope. The bias stress measurement was carried out on the Advanced Research Systems DE-204 temperature controlled stage.

Device Simulations

Device simulations were carried out using Silvaco Atlas. Atlas solves Poisson's equation, the charge carrier continuity equations and the charge transport equations [1]. SGT structures were simulated with a barrier inhomogeneity inserted into the Schottky source contact. The barrier height of the source was fixed as $\Phi_B^0 = 0.5$ eV, except at the inhomogeneity where the barrier height was $\Phi_B^0 - \Delta$. Only inhomogeneities with a barrier height lower than

Φ_B^0 were considered, as higher barriers would not contribute significantly to the current. Hence, the value of Δ was varied from 0, a homogeneous source, to 0.3 eV. Inhomogeneity position with respect to the drain end of the source edge was varied, such that $P = 0$, 10, 100, 1000, 4000 nm. The inhomogeneity width was also varied $L_0 = 3$, 10, 30 nm. Unless specified, the source length, S, and channel length, L, were fixed such that $S = 5$ μm and $L = 2$ μm. The IGZO thickness was varied such that $H = 10$, 20, 30, 50, 100 nm. The default Atlas model for IGZO was used [2]. The dielectric was SiO_2 and the dielectric thickness was fixed at 100 nm. The length of the drain was fixed at 1 μm and the gate overlapped the entirety of the source, drain and channel. The channel width was fixed at 1 μm.

XPS Measurements

XPS measurements were carried out using an Axis Ultra Hybrid (Kratos, Manchester UK), run at 10 mA emission with 15 kV bias. A charge neutraliser was used to remove any differential charging effects. The base pressure of the instrument was 10^{-8} mbar. The survey scan and the high resolution scans were run at 80 eV and 20 eV pass energies, respectively. High resolution scans were carried out on two ranges of interest, around the O 1s and Pt $4p_{3/2}$ peaks and the Pt $4f_{5/2}$ and $4f_{7/2}$ peaks, as well as the C 1s peak. The analysis was carried out with CasaXPS software. The binding energies were calibrated against the adventitious carbon peak at 284.8 eV. After calibration, the spectra were corrected by background subtraction. The spectra were fitted with a Gaussian-Lorentzian product formula, with the exception of Pt $4f_{5/2}$ and $4f_{7/2}$ peaks, which were fitted with a Lorentzian asymmetric

line-shape.

Supplementary Text

XPS

To understand why increasing the sputtering power of Pt in the presence of oxygen leads to greater barrier inhomogeneity, X-ray photoelectron spectroscopy was carried out on Pt films deposited under different conditions. Three Pt films were deposited on clean SiO_2-Si wafers, one in Ar at 60 W, one in 3% O_2/Ar at 60 W and one in 3% O_2/Ar at 40 W. The XPS results are shown in Fig. 1e.

When the Pt film was deposited in an Ar atmosphere the Pt $4p_{3/2}$ peak dominates over the O 1s peak and the Pt $4f_{5/2}$ and $4f_{7/2}$ peaks can be fitted with single asymmetric plots. There is also no metal oxide component to the O 1s peak, hence it can be concluded that the film contains a negligible quantity of oxygen, as expected.

Sputtering Pt in 3% O_2/Ar led to the emergence of a peak around 530 eV, which is associated with metal oxide. At 60 W, the ratio of the O 1s to the Pt $4p_{3/2}$ peak areas was about 1:4. At 40 W, the ratio increased to around 4:5, indicating increased oxidation. Furthermore, due to the formation of Pt oxides, the Pt $4f_{5/2}$ and $4f_{7/2}$ peaks shift to the left and require additional components to achieve a fitting. The peaks shift further to the left when the sputtering power is lowered from 60 to 40 W. The left shift indicates increasing oxidation, with peaks attributed to PtO, PtO_2 and high oxygen content Pt making increasingly large contributions as the sputtering power

is lowered [3].

The XPS results help to explain the effects of sputtering power and oxygen content upon the behaviour of Schottky diodes and SGTs shown in Figs. 1f and g. The increased oxidation at lower sputtering power can be attributed to the longer deposition time allowing for the inclusion of more oxygen in the film and therefore the Pt-IGZO interface. Hence, more In^{3+} is reduced when higher sputtering powers are used, leading to a greater density of lower barrier regions and a higher reverse current in the diodes. As a result, increasing power causes an increase in the on-current and reduction in turn-on voltage of the source-gated transistors.

Inhomogeneity Position

The contribution of a lower barrier region to the current is strongly dependent on its distance from the edge of the source nearest the drain, P. As shown in the output curves in Figs. S4a, b and c, the nearer the inhomogeneity is to the drain end of the source, the greater I_D is. In Fig. S4c, the current grows exponentially prior to saturation in all cases except when the inhomogeneity is at the edge of the source, i.e. $P = 0$ nm. At the edge, the inhomogeneity cannot be pinched off and no saddle point can form in the conduction band. Under these circumstances there is no voltage dependence of the effective barrier height and no exponential growth of current. The reason that the current is so strongly dependent on the position is because of the lateral resistance beneath the source. The further from the source edge the lower the potential at the interface. Thus, regions further from the source edge are less reverse-biased and so give a smaller current. As the inhomogeneity

dominates the current, the further from the source edge it is, the lower the total current from the source.

For similar reasons, the output impedance is also strongly dependent upon the position of inhomogeneity. When the device saturates at the source the potential at the semiconductor dielectric interface beneath the source is fixed and independent of V_D, except in the front 200 nm or so of the source. Hence, inhomogeneities within 200 nm of the source edge are only the ones that are affected by V_D in saturation, making them limiting factor for the output impedance, and therefore the intrinsic gain. Furthermore, as inhomogeneities far from the source edge increase the current contribution of this region of the source, they are likely to benefit geometrical tolerance and temperature dependence of the SGT [4].

SGT Theory

This theory applies for the high-gain condition only, i.e. the effects of the saddle points can be considered negligible, due to the semiconductor being made sufficiently thin or otherwise. In reverse bias, the current from the source is diffusion limited [5], therefore:

$$J_V(x) \approx q\mu_n N_C \mathcal{E}_M(x) \exp\left(-\frac{\Phi_B}{kT}\right)\left[1-\exp\left(-\frac{qV_{int}(x)}{kT}\right)\right] \qquad (1)$$

where $J_V(x)$ is the vertical current density from the source at position x, q is the fundamental charge, μ_n is the electron mobility in the semiconductor, N_C is the effective density of states in the conduction band, $\mathcal{E}_M(x)$ is the electric field at the Schottky interface, Φ_B is the barrier height, $V_{int}(x)$ is the potential at the semiconductor-dielectric interface at position x, k is the Boltzmann constant and T is the temperature. The band diagram is

described in Fig. S5a. At position x, $\mathcal{E}_M(x) \approx \frac{\phi_B^0 + V_{int}(x)}{H}$, where ϕ_B^0 is the mean barrier height potential ($\phi_B^0 = \Phi_B^0/q$) and H is the thickness of the semiconductor. Thus,

$$J_V(x) \approx q\mu_n N_C \frac{\phi_B^0 + V_{int}(x)}{H} \exp\left(-\frac{\Phi_B}{kT} \right) \tag{2}$$

When $V_D >> \phi_B^0$, we assume that the majority of current injection occurs where $V_{int} >> \phi_B^0$. Hence, the resistivity at position x can be given by:

$$\rho_V \approx \frac{V_{int}}{J_V} \approx \frac{H}{q\mu_n N_C \exp\left(-\frac{\Phi_B}{kT} \right)} \tag{3}$$

If we assume that $V_D << (V_G - V_T)$, where V_G is the gate voltage and V_T is the threshold voltage of the SGT, then beneath the source the resistance along the semiconductor-insulator interface is given by:

$$R_L \approx \frac{1}{\sigma_{ch}} = \frac{1}{q\mu_n N_{ch}} \approx \frac{1}{\mu_n C_G (V_G - V_T)} \tag{4}$$

where σ_{ch} is the conductivity of the channel, N_{ch} is the electron density in the channel, C_G is the capacitance per unit area of the gate dielectric and V_T is the threshold voltage of the SGT. Therefore, the effective source length is [6]:

$$L_{eff} = \sqrt{\frac{\rho_V}{R_L}} = \sqrt{\frac{HC_G(V_G - V_T)}{qN_C \exp\left(-\frac{\Phi_B}{kT} \right)}} \tag{5}$$

The threshold voltage of the SGT is given by

$$V_T = V_{T-TFT} + \frac{C_S + C_G}{C_G} \phi_B^0 \tag{6}$$

where C_S is the capacitance per unit area of the semiconductor and V_{T-TFT} is the threshold voltage of the semiconductor channel. In the linear regime,

where V_D does not cause full depletion of the semiconductor beneath the edge of the source (Fig. S5b), the current can be estimated in two different circumstances. If the source length $S >> L_{eff}$:

$$
\begin{aligned}
I_{lin} &= W L_{eff} J_V \\
&= W \sqrt{\frac{q N_C C_G (V_G - V_T) \exp\left(-\frac{\Phi_B}{kT}\right)}{H}} \\
&\quad \times \mu_n (\phi_B^0 + V_D) \left[1 - \exp\left(-\frac{qV_D}{kT}\right)\right]
\end{aligned} \tag{7}
$$

When $L_{eff} >> S$

$$
\begin{aligned}
I_{lin} &= W S J_V \\
&= W S q \mu_n N_C \frac{\phi_B^0 + V_D}{H} \exp\left(-\frac{\Phi_B}{kT}\right) \left[1 - \exp\left(-\frac{qV_D}{kT}\right)\right]
\end{aligned} \tag{8}
$$

Similarly, in the saturation regime, based on a series capacitance model the source saturation voltage is

$$
V_{Dsat} \approx \frac{C_G}{C_S + C_G}(V_G - V_T) \tag{9}
$$

thus, if the source length $S >> L_{eff}$:

$$
\begin{aligned}
I_{sat} &= W L_{eff} J_V \\
&= W \sqrt{\frac{q N_C C_G (V_G - V_T) \exp\left(-\frac{\Phi_B}{kT}\right)}{H}} \\
&\quad \times \mu_n \left[\frac{C_G}{C_S + C_G}(V_G - V_T) + \phi_B^0\right]
\end{aligned} \tag{10}
$$

When $L_{eff} >> S$

$$
\begin{aligned}
I_{sat} &= W S J_V \\
&= W S q \mu_n N_C \frac{\frac{C_G}{C_S + C_G}(V_G - V_T) + \phi_B^0}{H} \exp\left(-\frac{\Phi_B}{kT}\right)
\end{aligned} \tag{11}
$$

If considering image force lowering:

$$\Phi_{IFL} = q\sqrt{\frac{q\mathcal{E}_M}{4\pi\epsilon_0\epsilon_s}} = q\sqrt{\frac{q\left[\phi_B^0 + \left(\frac{C_G}{C_S + C_G}\right)(V_G - V_T)\right]}{4\pi\epsilon_0\epsilon_s H}} \tag{12}$$

Based on recent research, the Pt-IGZO interface is not abrupt [7]. There is a transition region where Pt clusters are encapsulated by In, which may lead to interfacial states. Such interfacial states are can lead to a barrier lowering effect with a magnitude of $\alpha q\mathcal{E}_M$ [8]. Similar trends can also be attributed to tunnelling or the electric field penetrating the metal [8]. Combining these effects, the effective barrier at the source is

$$\Phi_{B,eff} = \Phi_B^0 - \Phi_{IFL} - \alpha q\mathcal{E}_M \tag{13}$$

By substituting $\Phi_{B,eff}$ for Φ_B in the equations for I_{lin} and I_{sat} we arrive at the formulae for fitting the *I*-*V* characteristics in Figs. 2i and j.

Self-Heating

The high current densities that pass through inhomogeneities or the source edge may lead to concerns about self-heating degrading the SGT characteristics after long-term operation. Device simulations have previously indicated that self-heating is not a major hurdle to device application and can be alleviated by increasing the source length or using a field plate [9]. A field plate is an additional capacitance at the front edge of the source that moves the region of high electric field away from the source edge. Further work may be required on this issue, but self-heating does not appear to be a significant issue for the devices measured in fabricated in this work, which demonstrate excellent stability over long measurement times.

Supplementary Figures

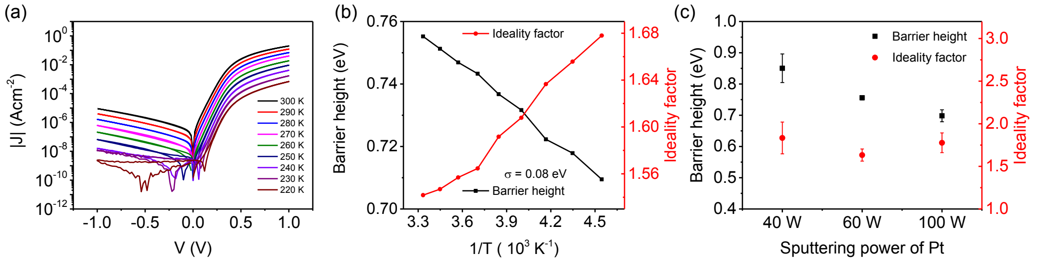

Figure S1: (a) $|J|$-V curves of the Pt-IGZO diodes for different temperatures from 220-300 K. Pt was deposited at 60 W in 3% O_2/Ar. (b) Barrier height and ideality factor against $1/T$ for the device in (a). The temperature dependence of the barrier height indicates the presence of barrier inhomogeneities. The standard deviation from the mean barrier height is $\sigma = 0.08$ eV. (c) Barrier height and ideality factor as a function of Pt deposition power. Error bars show the standard deviation from the mean.

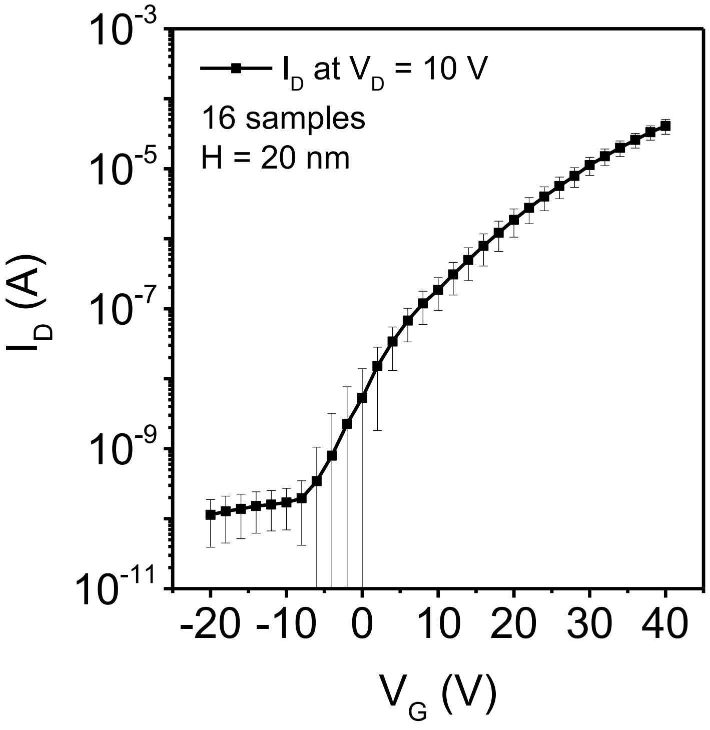

Figure S2: Statistical data for 16 SGT transfer curves with an IGZO thickness of 20 nm and Pt deposited at 60 W in 3% O_2/Ar. Error bars show the standard deviation from the mean.

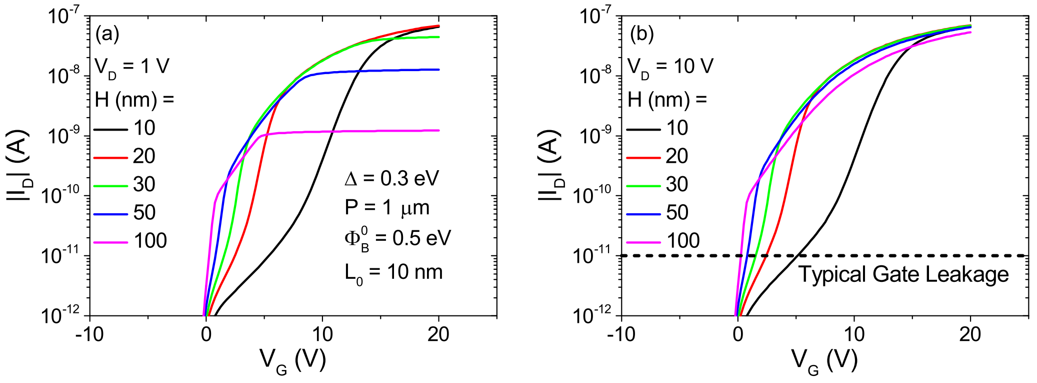

Figure S3: Simulated transfer curves for SGTs with a barrier inhomogeneity at the source for $V_D = 1$ V (a) and $V_D = 10$ V (b). IGZO thicknesses of 10, 20, 30, 50 and 100 nm were simulated. The inhomogeneity had a magnitude $\Delta = 0.3$ and the inhomogeneity was 1 μm from the drain end of the source. The results reflect the experimental results shown in Figs. 1i and j. Similar results can be seen for different values of P and Δ.

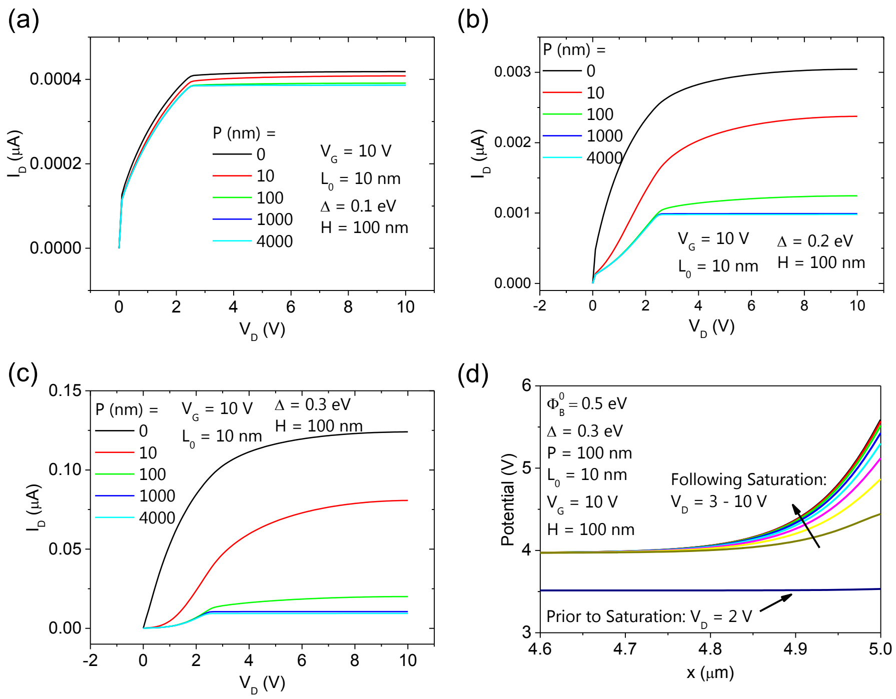

Figure S4: The effects of inhomogeneity position upon the characteristics of an SGT with a 100 nm thick semiconductor layer. The inhomogeneity is 10 nm wide and $V_G = 10$ V. (a) Output curves for $\Delta = 0.1$ eV. (b) Output curves for $\Delta = 0.2$ eV. (c) Output curves for $\Delta = 0.3$ eV. (d) Potential at the semiconductor-dielectric interface for different values of V_D. The source edge is at $z = 5$ μm.

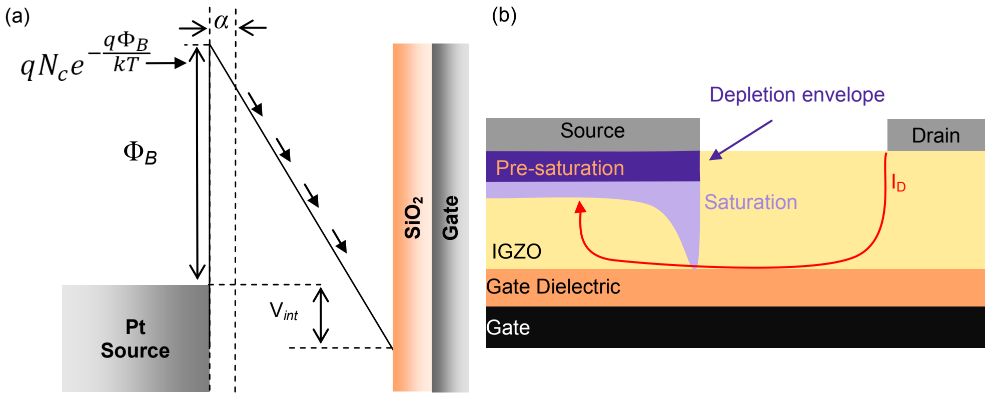

Figure S5: Explanation of source-gated transistor theory. (a) Conduction band between the source and the semiconductor-dielectric interface showing the mechanism of current injection. (b) Structure of a source-gated transistor showing the shape of the depletion region before and after current saturation.

References

[1] Silvaco, Inc. (2016) *Atlas User's Manual.*

[2] Fung TC, et al. (2009) Two-dimensional numerical simulation of radio frequency sputter amorphous In–Ga–Zn–O thin-film transistors. *J. Appl. Phys.* 106(8):084511.

[3] Parkinson C, Walker M, McConville C (2003) Reaction of atomic oxygen with a Pt(111) surface: chemical and structural determination using XPS, CAICISS and LEED. *Surf. Sci* 545(1-2):19–33.

[4] Sporea R, Overy M, Shannon J, Silva S (2015) Temperature dependence of the current in schottky-barrier source-gated transistors. *J. Appl. Phys.* 117(18):184502.

[5] Valletta A, Mariucci L, Rapisarda M, Fortunato G (2013) Principle of operation and modeling of source-gated transistors. *J. Appl. Phys.* 114(6):064501.

[6] Schroder DK (2006) *Semiconductor Material and Device Characterization.* (John Wiley & Sons Inc., New Jersey, U.S.A.), 3rd edition.

[7] Flynn BT, Oleksak RP, Thevuthasan S, Herman GS (2018) Interfacial chemistry induced modulation of Schottky barrier heights: in-situ measurements of the Pt–amorphous indium gallium zinc oxide interface using X-ray photoelectron spectroscopy. *ACS Appl. Mater. Interfaces* 10(4):4333–4340.

[8] Rhoderick EH, Williams RH (1988) *Metal-Semiconductor Contacts, Monographs in Electrical and Electronic Engineering No. 19.* (Clarendon, Oxford).

[9] Sporea RA, Burridge T, Silva SRP (2015) Self-heating effects in polysilicon source gated transistors. *Sci. Rep.* 5:14058.
